# Book Reviews

**Published:** 1822-12

**Authors:** 


### MEDICAL AND SCIENTIFIC BIBLIOGRAPHY.

Vexat censura . . . . . . Corvos.

Art. I.—*A new View of the Infection of Scarlet Fever, &c. &c.* By Wm. Macmichael, m.d. f.r.s. Fellow of the Royal College of Physicians, and Physician to the Middlesex Hospital. London, 1822.

The professed object of this little work is to recommend that children and others, who may not have had the scarlatina, should be exposed to its influence during the prevalence of auspicious epidemics. From this advice (which the author asserts, with justice, to be new,) we expected to find him holding, as already ascertained on incontrovertible grounds, that a mild scarlatina always gave rise to an equally mild form of the disease; we were, therefore, not a little surprised to find, at p. 60, that "it has long been indisputably established that the slightest of all eruptive fevers, namely, the simple scarlet fever, and that most fatal disease the malignant sore-throat, are varieties only of the same disease, and *proceed from the same contagion.*" Few parents, we suspect, who read this passage, will be much influenced by the view Dr. Macmichael has taken in the latter part of the pamphlet; and, for our own part, we must acknowledge we could not feel comfortable in urging the exposure of their children to a contagion, which, however mild in appearance, might *possibly* communicate a malignant and fatal complaint. Besides this ostensible object of the work, various cursory remarks are made on Pellagra, Ergot and Malaria, Typhus, Measles, and Small-pox; and some interesting observations on the nature of those causes which occasionally lead to the rapid and extensive propagation of disease, constituting an epidemy. These have generally been regarded as depending on certain peculiarities of atmosphere, &c. of a nature too subtle for our detection: Dr. Macmichael thinks it would be better to have in view the "peculiar state or constitution of body into which a great number of people are brought, by having been subjected to the operation of the same physical and moral causes." This *constitutio epidemica* depends upon the influence of weather, diet, occupation, state of mind, and other causes, the operation of which is often first manifested by the occurrence and rapid increase of certain diseases. These remarks of Dr. M. are important, and open a field for much interesting investigation; which, however, it would be foreign to the object of this department to prosecute further.

Art. II.—*An Address to Parents on the present State of Vaccination in this Country, &c.* By A Candid Observer.

A "candid Observer" is one of the most uncandid persons imaginable. The title he assumes is obviously intended to convey the impression that he is not one of the profession, but rather steps forward in consequence of some want of candour on their part; by which it is hoped the mothers of families, and reading country gentlemen, may be seduced into a perusal of his book. After they have been thus unwittingly drawn into the snare, by some introductory finessing about the "faculty," he most uncourteously turns upon his readers, and overwhelms them with facts, histories, authorities, calculations, and the whole train of offensive weapons usually employed by writers who are conscious of having the best side of the argument. There are two circumstances which perfectly convince us that this is not the production of an unprofessional man: first, he understands the subject thoroughly; and, secondly, he speaks well of the "doctors." Whether others who read it may draw the same conclusion as to its author, may be a question; but no one, we are sure, will deny that he is impartial in his statements, and convincing in his inferences. It is true there are no new doctrines advanced, nor any pretensions to original views; but the work is thereby better calculated to fulfil the object of the writer, which is literally to give a fair estimate of the protection afforded by vaccination against small-pox, and of the object a mother ought *reasonably* to have in subjecting her child to cow-pox. It is a book about which medical men are likely to be questioned in families where they visit, and we therefore advise them to read it. We think the pamphlet calculated to do good, and therefore we have thus early noticed it. We cannot attempt to enter into the merits of so extensive a question at present; and our object is, therefore, rather to excite curiosity than to gratify it. "The work (as we were lately informed by a learned author) is to be had at the booksellers."

---

ART. III.—*Observations (from Experience) on the Aid obtained in various Diseases, particularly those incidental to Tropical Climates, by the external Application of the Nitro-muriatic Acid in a Bath; with several Cases wherein it has been used by the Author with great utility. To which is added, the present most approved Mode of mixing the Acids and preparing the Bath.* By PHINEAS COYNE, Member of the Royal College of Surgeons, London, and late of the Honourable East India Company's Service. 8vo. pp. 144. John Warren, London, 1822.

"How prone to doubt, how cautious are the wise."

SUCH is the full and elaborate account of the work before us, as set forth in the title-page, which we have given at length, as it is the only portion of the work with which we mean to take a similar liberty. HORACE advises a poet to keep his effusions nine years before he commits them to the public scrutiny; and this we imagine to have been in order that they might be revised and corrected. Mr. COYNE has deemed one-third of this period sufficient for his observations; and we are so far from finding fault with this, that we would recommend it to the imitation of others, but that we would rather offer advice which there was some chance of being followed. Yet our commendation of Mr. Coyne's forbearance is considerably qualified by finding that this delay was not occasioned by any motive of deference to the opinion of the great Roman satirist, and that the information of his having kept it by him so long is given us because it is necessary "to account for many passages, which otherwise would appear inexplicable, if not unintelligible; as also the names of persons now *no more,* and others whose situations in life are so changed that, even within the short space of three years, they could scarcely be recognized." The reason of this proceeding was, that Dr. Scott had mentioned to our author his intention of publishing remarks upon the effects of the nitro-muriatic acid bath: after this information, Mr. Coyne, although his book was actually printed, withheld it from the public, and asks, "Would it not have been the very acmé of presumption in me to forerun, by any publication on the subject, such a man?"

The Introduction, besides this information, contains some desultory remarks on Cholera, in which he condemns bleeding and recommends mercury and the bath; but his opinions are deprived of much of their weight, when we find him talking of "what he could glean by conversing with gentlemen lately returned from India." We strongly suspect, from his manner of writing, that he has not witnessed the practice of blood-letting, but condemns it on theoretical principles. The preparation of mercury recommended is the oxymuriate, from a quarter of a grain to one grain for a dose, repeated every second, third, or fourth hour, according to the urgency of the case! combining it with opium, in doses of two, three, or four grains.

The principal object of this work is to show that the nitro-muriatic acid bath was received with much distrust in this country; that it was from the first viewed with prejudice, and condemned by most practitioners untried: all which we think is correct; and, farther, that the prejudice is not likely to diminish, because no satisfactory evidence of its utility has been laid before the public by any of its supporters. Even in the recent work of Mr. Wallace, effects are attributed to this remedy which appear to us to have arisen from other causes. With regard to the trial it has received, we would observe, that medical men are not to be censured who decline trying upon their patients every ephemeral specific that may be proposed; and that it is only in public institutions, where the subjects of experiment are numerous, under control, and seen by many, that trials of this kind can properly be made. An extensive, and we believe in every respect fair, opportunity of judging of this remedy was afforded at the York Hospital, and the result was unfavourable to the acid. Our author, it is true, objects to the manner in which these trials were made, especially with regard to the proportions of the acids. "That Mr. Goutherie's experiments were extremely incorrect, he will himself (I have not the slightest doubt,) admit, when it is known that the proportions of the acids which he used (three parts of nitric to one of muriatic,) are directly the opposite of those which I have always found best,—namely, three parts of muriatic to one of nitric." (p. 35.) But the words, that "no dependence can be placed on *any particular proportion,*" show that the proportion was varied in different experiments. Mr. Coyne is anxious to do away the effect of this unfavourable result at the York Hospital, among other reasons, because "Mr. Goutherie (Guthrie,) ranks so high in his profession, that his *ipse dixit* would tend more to the condemnation of the remedy than all the theoretical reasoning that could be brought against it." This fear is groundless: no man can rank so high in science or reputation as to make his *ipse dixit* pass current as authority, unless it be founded on observation; and if so, it deserves to be admitted.

These trials, however, were not deemed sufficient, and fresh experiments were made by the direction of his Royal Highness the Duke of York: the fairness and candour with which these were conducted are beyond all dispute, and are acknowledged by Mr. Coyne. "When I heard this order," says he, "and saw the trials were made with the utmost ingenuousness, I confess that I was very highly gratified." One of the surgeons in charge was "Mr. M'Cloud (Macleod), whose scientific observations on the different diseases afforded us the greatest assistance." Mr. C. thinks that "extraordinary good effects" resulted from the use of the bath in these cases; but Mr. Macleod, we know, from personal communication with him, was led to a different conclusion.

Next follow some observations upon the connexion between phthisis and hepatic diseases, a subject which has been fully and ably illustrated by Dr. Wilson Philip.

Seventeen cases are detailed, in all of which, except one, it proved signally useful, producing either complete cure or great amendment. The complaints were (or were supposed to be,) scrofula, hydrocephalus, herpes, consumption, epilepsy, diseased liver, "atrophy," dropsy, diarrhœa, ulcers of the extremities, and ulcers of the throat with nodes. In fifteen of the cases other remedies are incidentally mentioned, but not regarded as contributing in any considerable degree to the cure. Of the fourteenth, we are told that the patient "commenced the nitro-muriatic bath, (every other remedy having been discontinued;)" and immediately after an exception is made in favour of "a wine-glassful of chamomile tea, cold, twice a-day, and a small quantity, when necessary, of the common aperient medicine of the hospital."

We are decidedly of opinion that this remedy has been viewed with prejudice: we believe it is not, as has been often asserted, totally useless; but we do not think the indications it is capable of fulfilling have been distinctly ascertained; and the list given above of the diseases in which Mr. C. thought it of use, is too extensive to afford much assistance.

It is the misfortune of all new remedial means proposed, and of none more than the nitro-muriatic acid bath, to be extolled above their deserts; the obvious tendency of which is to defeat the views of their supporters, and bring them into discredit, perhaps greater than they deserve. In addition to the list of diseases above given, we are informed, in the Preface, on the authority of some "skilful medical friends in Ireland," that the remedy is useful in other complaints,—'more particularly, in typhus its assistance has been deemed invaluable," "and found to be a complete remedy in marasmus, infantile remittents, and tabes mesenterica." Mr. Coyne will forgive our reminding him of his motto—"How prone to doubt, how cautious are the wise!"

ART. IV.—*An Inquiry into the Action of Mercury on the living Body.* By JOSEPH SWAN, Member of the Royal College of Surgeons, and Surgeon to the Lincoln County Hospital. pp. 30. London, 1822.

THE attention we have already paid to, and the notice we have taken of, Mr. SWAN's former productions, will, we trust, sufficiently show our estimation of that gentleman's zeal, industry, and talent; and it is because we feel a sincere regard for his character, that we are induced to say a few words respecting his little pamphlet on the action of Mercury; for we were grieved to see Mr. Swan risking his reputation, and sinking to the level of a mere pamphleteer, by publishing so crude, ill-founded, and ill-supported an argument as that which appears at the head of this article.

It is true that Mr. Swan has really paid great attention to the nervous system; but it is not therefore incumbent upon him to refer the action of every medicine to the medium of the nerves, nor all the phenomena of disease to their mobid condition; and he has thought but little upon the subject if he has not already discovered how much he has omitted, in the course of his argument, that favours his own views, and how entirely he has overlooked every thing that makes against him. We should wish to know of Mr. Swan what becomes of mercury, either rubbed into the skin or taken into the stomach? He says, that it is not to be detected in the secretions: how, then, is it got rid of? and by what process independently of absorption? If it passes off by stool, how is that to be explained, without the aid of the absorbents, in the case of mercurial frictions upon the surface of the body?

To what purpose does Mr. Swan tell us that there is no evidence of the absorption of mercury into the constitution? Does not that medicine uniformly accelerate the circulation, and increase all the secretions? and is not this effected through the medium of the circulation? Grant that the nerves are primarily acted upon, what inference is to be drawn from that circumstance, if proved; and how is it proved in the pamphlet before us?—by the dissection of two bitches, who have the submuriate of mercury administered to them for several successive days, until they die. The result of the dissection is, that the par vagum, in each instance, was very vascular. Is not this always the case? and were not all the mucous surfaces very vascular also? and what was the condition of the stomach and the intestinal canal? Finally, what was the comparative vascularity of the nerves of a sound dog, and one killed by mercury?

Mr. Swan seems to think that the nitro-muriatic acid bath produces its effect upon the salivary organs through the medium of the nerves also. Now, we think that this instance rather makes against his argument than for it, because the effect of these baths is only produced by their continued use; and the same result is also found occasionally to follow the exhibition of large doses of the carbonate of iron, and some other medicines, when continued for a length of time. Mr. Swan also omits to mention the condition of the blood when the system is fully under the use of mercury; a circumstance well known to the commonest observer. Neither is it true that mercury has not been detected in the human body: a case in point is recorded in the fifth volume of the Memoirs of the Medical Society of London, in which paper will be found references to many other writers who have recorded similar facts.

In the Proëmium to the forty-sixth volume of this Journal, by our learned predecessor Dr. HUTCHINSON, will be found still stronger and more conclusive evidence on this point. Professor AUTENREITH has asserted that he found mercury under such circumstances; the authorities of FALLOPIUS, MEAD, VAN SWIETEN, BONETUS, FOURCROY, &c. all go to prove the same fact; and therefore, in the words of Dr. Hutchinson, "we must infer, not that mercury is *not* absorbed and carried into the circulation, but that, after being thus received, it is quickly evacuated by the emunctories; and that the symptoms of its action will continue for a considerable time after it has ceased to be present in the body."

Among the omissions in favour of his own views, Mr. Swan has entirely overlooked the deleterious effects of mercurial vapour, all purely nervous; as well as that peculiar state of the constitution, called by Mr. PEARSON "Erithesmus," and which occasionally destroys life in an instant, upon the most trifling motion or exertion. Dissection does not, in this instance, explain the immediate cause of death; but it is remarkable that none of these diseased actions arising from the operation of mercury take place when it exerts its proper influence upon the constitution, and that, in this latter instance, so far from the patient appearing distressed, or overpowered by the medicine, the spirits are usually exhilarated, the pulse increased in frequency and strength, and all the secretions are augmented. In fine, the subject here discussed by Mr. Swan in the course of a few pages, and those by no means closely printed, is one of the most difficult and delicate that can be proposed to the physiological enquirer, and we should run the risk of writing an essay longer than the work itself, if we were to say all that might be said in answer to our author's observations; but as Mr. Swan holds out a promise of pursuing his inquiries upon a more extended scale, we shall pass on to the practical inferences which he draws from the view he has taken of the action of this powerful medicine. We have no material objection to urge against what he has said relative to the too profuse exhibition of mercury by mothers, or other would-be Lady Bountifuls: we believe it to be a practice fraught with evil in the hands either of male or female pretenders to medical knowledge; but we cannot go the length of supposing, with some, that the prevalence of scrofula is owing to this in any considerable degree, because that disease was long known by the name of the English malady, before mercury was ever administered excepting *for one complaint;* and we might as well pretend that the increase of strumous complaints is owing to the abolition of that laudable and scientific remedy, the royal touch.

We beg that Mr. Swan will believe that, in what we have urged above, we have been solely guided by a sincere regard for him, and for that reputation which he has so honourably acquired, and which we feel sorry that he should run any hazard of diminishing, or losing, by the publication of works hastily put together, not sufficiently digested, and certainly not imperatively called for by any temporary circumstances, which in some instances may palliate, though not entirely justify, the publication of unconnected hints or hasty surmises.

---

Art. V.—*Observations on the Effects of Mercury on the Organs of Hearing, and the improper Use of it in Cases of Nervous Deafness.* By W. Wright, Surgeon-Aurist to her late Majesty Queen Charlotte, &c. pp. 24. London, 1822.

Mr. Wright informs us, in an introductory address, that this little pamphlet is only the precursor of a larger work, in which all the methods of curing deafness, or diseases of the ear, either in this country or on the continent, including a condensed translation of the work of M. Itard, will be detailed: this, therefore, may be designated a warning against the use of mercury in cases of nervous deafness, a practice introduced (into this country, at least,) by Mr. Saunders, and which the author before us most unequivocally condemns. We agree with Mr. Wright in the greater part of his remarks upon this subject; but we think that he has, perhaps, carried his general condemnation of mercury as a remedy too far; and we are not satisfied always with the terms which he has employed in the course of these observations: for example, at page 10, he says,

"Where syphilitic ulceration exists in the throat, or in cases of inflammation of the pharynx from this cause, mercury may be indispensable. Yet, upon this vague and uncertain idea of its usefulness in nervous deafness, which even the modern advocate of the practice declared was erroneous, the most delicate females, as well as persons of all ages, have been indiscriminately saturated with mercury; and the public have been favoured, from time to time, with accounts of cures of persons by this method, who seem to have neither a local habitation nor a name; for the initials only are given, which, with the exception of three or four, comprize all the letters of the alphabet, some of which, to add to their importance, are graced with titles. *This publication of anonymous cases carries no conviction to the mind of a person of common intellect;* for he has before him only the *ex-parte* statement of a person who is interested in proving to the world, as far as his *ipse dixit* will do so, that he is superior to every other person in the treatment of a peculiar species of disease. As far as concerns the profession, this conduct is illiberal; and, as concerning the interest of the party adopting it, the plan is ill-judged; for the public, and the respectable part of the profession, look upon most of these things as mere advertisements, and the veriest empiric will surpass any practitioner of real merit by the unblushing effrontery with which he will fabricate cases, and even introduce the names of some of the nobility as his patrons or patients, who never heard of him, but neglect to expose the imposition, lest they should be giving importance by noticing the falsehood."

Now, towards the end of the pamphlet, we find our author detailing several cases in favour of his own opinions, all of them *anonymous*, and not narrated with much attention to "local habitation" either, or to the particular circumstances of the cases themselves.

We shall wait patiently until Mr. Wright's promised work makes its appearance, and hope then to give an extended analysis of his opinions and practice in diseases of the ear,—a class of complaints very little understood, and the treatment of the majority of which may be called almost entirely conjectural. In the mean while, we think this little pamphlet will probably be serviceable in checking that modern species of empiricism, which has immediate recourse to mercury for the removal of all ill-understood, or unexplained, forms of disease.

---

ART. VI.—*The Way to preserve good Health, &c.; together with a Treatise on Domestic Medicine, &c.* By ROBERT THOMAS, M.D.

WE have never yet been convinced that medicine was a science of such easy attainment, as to be acquired without much study, both in the closet and at the bed-side of the patient. If this be so, what good purpose is fulfilled by any system of domestic medicine, in a country where every village now possesses one or more regularly-educated practitioners? We fear, however, the evil is not merely negative, but frequent and serious. The mother of a family, having taken the trouble to read, is unwilling that her learning should be entirely lost, and needs must practise upon her children. The author before us asserts, in his Preface, that "those who may suppose their interest likely to be somewhat affected by a work of this nature, may perhaps raise objections:" but, so far as medical men are concerned, the fact is diametrically opposite to what Dr. Thomas insinuates; for we are convinced the more his, Dr. Buchan's, or any other system of Domestic Medicine, is perused in families, the more professional advice such families will require from their attendants, to remedy mistakes, and cure, if it be not too late, those diseases which, though mild in their nature, and simple in their treatment, have become exasperated by bad management, or confirmed by neglect of proper means.

But Dr. Thomas may choose to state the question thus:—As there are already many Lady Bountifuls, and worthy clergymen who take the trouble to look after the bodies as well as the souls of their flocks, is it not better that they should have my book than any one which is inferior? Now, in this we agree with him; not intending the foregoing remarks as peculiarly applicable to him, who indeed has already shown himself to be a most industrious compiler,—and all systematic works can be little else than compilations. The one before us is the best of the kind we have met with, containing some useful remarks on diet and regimen, and having directions for changes of treatment corresponding to different climates,—a recommendation which few, if any, of the others possess. We would advise all who are not of the profession, if they would consult their own safety and comfort, to leave the perusal of medical books to medical men; but, if they *will* read them, then we recommend the "Treatise on Domestic Medicine" of Dr. Thomas.

